# From pathogenesis to treatment: the emerging role of ferroptosis in Parkinson’s disease

**DOI:** 10.3389/fimmu.2025.1709561

**Published:** 2025-11-26

**Authors:** Ruoxin Tu, Zhongyu Han, Hongbo Zhang, Xiaoye Jia, Tong Sun, Hanlin Liu, Jingxian Li, Mingke Tang, Shun Wang

**Affiliations:** 1 Institute of Acupuncture and Moxibustion, Heilongjiang Academy of Traditional Chinese Medicine, Harbin, China; 2 Institute of Nephrology, Zhongda Hospital, School of Medicine, Southeast University, Nanjing, China; 3 The Second Clinical Medical College, Heilongjiang University of Traditional Chinese Medicine, Harbin, China; 4 The Second Affiliated Hospital, Heilongjiang University of Chinese Medicine, Harbin, China

**Keywords:** ferroptosis, Parkinson’s disease, neuronal death, oxidative stress, lipid peroxidation, epigenetics

## Abstract

Parkinson’s disease (PD), the second most common neurodegenerative disorder worldwide, features gradual loss of dopaminergic neurons in the substantia nigra pars compacta (SNpc) along with pathological α-synuclein (α-syn) aggregation. Recently, emerging evidence has identified ferroptosis, an iron-dependent regulated cell death, as a pivotal factor in driving PD pathogenesis, with close associations to key mechanisms including α-syn protein aggregation, excessive oxidative stress, mitochondrial dysfunction, disturbances in iron metabolism, and activation of neuroinflammatory responses. This distinct mode of regulated cell death provides novel perspectives for understanding the underlying pathogenesis of PD. This review highlights the mechanisms of ferroptosis, its contribution to PD pathogenesis, evidence from animal models, and clinical advances in ferroptosis-targeted therapies. Moreover, we put forward the potential of ferroptosis in the early diagnosis and treatment of PD. A profound understanding of the ferroptosis-PD crosstalk provides a new perspective on neuronal vulnerability and holds promise for advancing novel treatments for this disabling disorder.

## 1 Introduction

Parkinson’s disease (PD) is the most common movement disorder and the second most prevalent neurodegenerative disease (1, 2). First identified by Dr. James Parkinson in 1817 and later termed “Parkinson’s disease” by Jean-Martin Charcot, PD is characterized by motor symptoms such as resting tremor, rigidity, bradykinesia, and postural instability, as well as non-motor manifestations including sleep disturbances and autonomic dysfunction (3, 4). Pathologically, PD is defined by the progressive dopaminergic neuronal loss in the substantia nigra pars compacta (SNpc) and the formation of intraneuronal Lewy bodies, cytoplasmic inclusions primarily comprising α-synuclein (α-syn) fibrils (5, 6). The underlying causes and pathological mechanisms of PD are still largely undefined, though factors such as α-syn aggregation, iron overload, neuroinflammation, and lipid peroxidation are recognized as key pathogenic factors in dopaminergic neurodegeneration (7, 8).

Given the incomplete understanding of PD’s underlying pathological mechanisms, current therapeutic strategies primarily offer symptomatic relief rather than halting disease progression. Levodopa, the most potent symptomatic therapy, markedly improves motor symptoms, yet it comes with considerable adverse reactions (9, 10). Other medications, such as dopamine receptor agonists and anticholinergics, also have limitations, including impulse-control disorders and various anticholinergic side effects (11, 12). Even deep brain stimulation, while effective for some motor symptoms, does not stop neurodegeneration (13). Thus, it is imperative to investigate new treatment avenues and their underlying mechanisms that can address the fundamental pathophysiology of PD and slow or stop neuronal loss.

**Figure 1:**
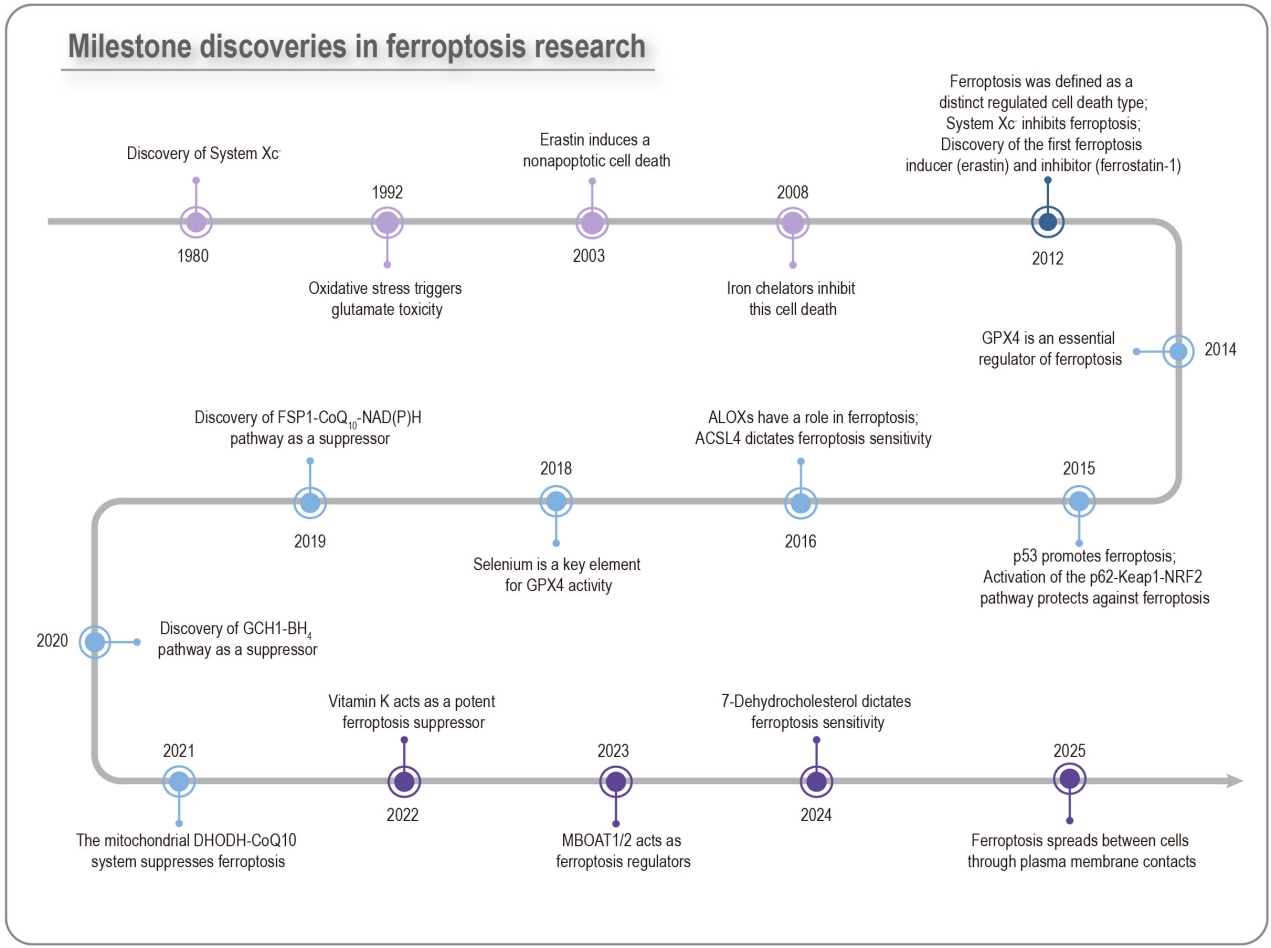
Milestone discoveries in ferroptosis research. The key ferroptosis-associated membrane protein xCT was identified as early as the 1980s. After more than two decades of investigation, the canonical ferroptosis inducers erastin and RSL3, which were found to trigger cell death in RAS-mutant BJELR cells, were reported in 2003 and 2008, respectively. However, it was not until 2012 that Dixon and colleagues formally defined this process as ferroptosis. Since then, ferroptosis has surged to the forefront of research, bringing multiple major advances.

As early as 1982, glutathione peroxidase 4 (GPX4) was reported to inhibit the accumulation of lipid hydroperoxides, thus preventing plasma membrane damage (14). In 1989, Murphy and colleagues described a neuronal death process resulting from cysteine scarcity, termed “oxytosis”, which was induced by glutamate inhibiting SLC7A11 (a component of the Xc^-^ system) and shared several characteristics with ferroptosis, including reactive oxygen species (ROS) accumulation and high lipoxygenase enzymatic activity (15, 16). In 2003, Dolma et al. observed that erastin induced a distinct form of non-apoptotic cell death in cancer cells with RAS overexpression, which was not inhibited by caspase inhibitors (17). In 2008, Yang et al. reported that RAS-selective lethal small molecule 3 (RSL3) also triggered a similar iron-dependent cell death (18). Concurrently, other studies showed that GPX4 deficiency induced lipid peroxidation and non-apoptotic cell death that could be averted by Xc^-^ system overexpression (19, 20). Based on these characteristics, in 2012, Dixon et al. officially named and defined this unique form of cell death “ferroptosis” as an iron-dependent and regulated process driven by lipid peroxidation (21), distinguishing it from other cell death modalities. Over the subsequent decade, ferroptosis has rapidly emerged as a prominent research field, yielding numerous remarkable advances (**Figure 1**).

Intriguingly, several key pathological features of PD align remarkably with the triggers and hallmarks of ferroptosis, including amplified lipid peroxidation, increased iron accumulation, suppressed expression of the cysteine-glutamate antiporter (system Xc^-^) and diminished glutathione (GSH) levels (22, 23). Studies have shown that in early PD patients with a disease duration of less than 4 years, iron accumulation in the SNpc occurs 4.1–9.6 years prior to the clinical diagnosis of PD and precedes SNpc neuromelanin (NM) loss—a marker of dopaminergic neuronal degeneration—by 2.6–3.7 years. Moreover, PD patients with elevated SNpc iron content exhibit a 35–40% reduction in dopamine transporter specific binding ratio in the sensorimotor putamen, the earliest and most severely affected striatal subregion, indicating striatal dysfunction (24). Meanwhile, α-syn aggregation significantly promotes iron-mediated lipid peroxidation. The basal level of lipid peroxidation in *SNCA* triplication neurons is approximately 1.5-fold higher than that in control neurons, and exposure to 10 nM α-syn oligomers further elevates lipid peroxidation levels (25). Given that oxidative stress is a well-established contributor to neuronal loss in PD, the iron-dependent and lipid peroxidation-driven nature of ferroptosis strongly suggests its potential involvement in PD pathogenesis (8, 26).

Consequently, exploring ferroptosis as a pivotal mechanism in dopaminergic neuron demise offers promising avenues for novel therapeutic interventions in PD. Preclinical studies have shown that ferroptosis-preventing therapies, such as the GSH precursor N-acetylcysteine (NAC), iron-chelating agents like deferoxamine (DFO) and deferiprone (DFP), and specific ferroptosis inhibitors like ferrostatin-1 (Fer-1), can alleviate neurodegeneration and improve motor symptoms in PD animal models (27–29). Ample evidence suggests that plant-derived compounds also modulate ferroptosis in PD, providing promising lead compounds for future drug development (30).

In this comprehensive review, we aim to summarize the multifaceted roles of ferroptosis in PD pathogenesis, elucidate the links between PD-associated pathogenic proteins and ferroptosis, and provide an overview of current clinical trials and the potential of ferroptosis in the early diagnosis and treatment of PD.

## 2 Ferroptosis in PD

**Figure 2:**
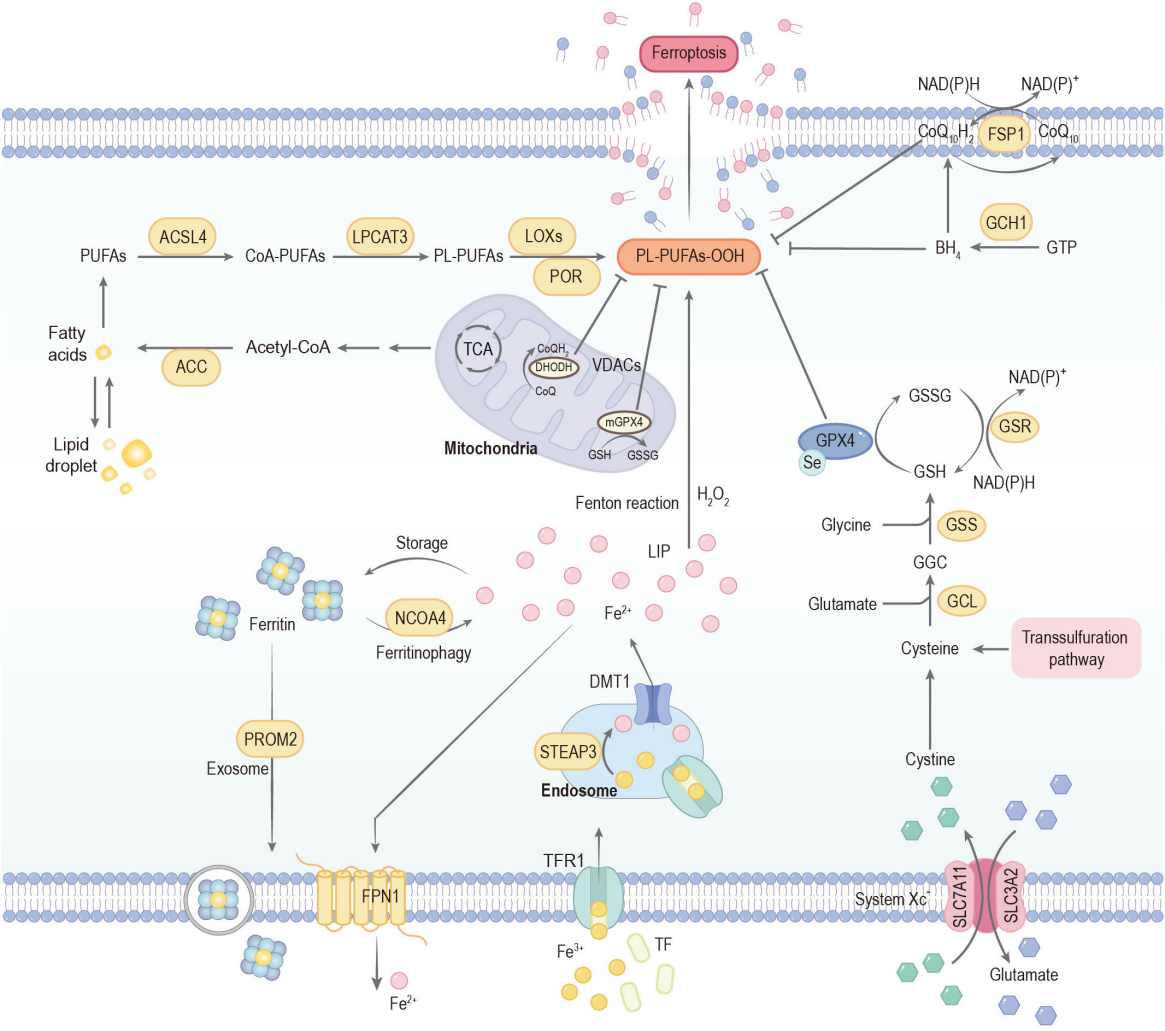
Mechanisms governing ferroptosis. Ferroptosis is predominantly driven by iron-dependent lipid peroxidation. Extracellular iron is primarily bound to transferrin (TF) and internalized into endosomes via transferrin receptor 1 (TfR1). Within the endosomal compartment, the acidic microenvironment maintained by proton pumps facilitates the reduction of Fe^3+^ to Fe^2+^, a reaction catalyzed by six-transmembrane epithelial antigen of prostate 3 (STEAP3). The reduced Fe^2+^ is subsequently transported into the cytoplasm through divalent metal transporter 1 (DMT1), thereby contributing to the formation of the labile iron pool (LIP). Cytoplasmic iron homeostasis is tightly regulated: excess Fe^2+^ can be exported by the membrane protein ferroportin (FPN1) or sequestered in ferritin for storage. Notably, ferritin can undergo selective autophagic degradation mediated by nuclear receptor coactivator 4 (NCOA4), a process termed ferritinophagy, which releases stored iron into the LIP and consequently increases cellular susceptibility to ferroptosis. Acetyl-CoA carboxylase (ACC)-mediated fatty acid synthesis or lipophagy-mediated fatty acid release induces the accumulation of intracellular free fatty acids, which fuels ferroptosis. Long-chain acyl-CoA synthetase 4 (ACSL4) and lysophosphatidylcholine acyltransferase 3 (LPCAT3) promote the incorporation of polyunsaturated fatty acids (PUFAs) into phospholipids to form phospholipid-polyunsaturated fatty acids (PL-PUFAs). These PL-PUFAs are highly susceptible to peroxidation, occurring through enzymatic pathways mediated by lipoxygenases (LOXs) and cytochrome P450 oxidoreductase (POR), as well as non-enzymatic free radical–driven reactions, ultimately promoting ferroptotic cell death. The cystine/glutamate antiporter (System Xc^-^) mediates the uptake of cystine in exchange for glutamate at a 1:1 ratio. Once transported into the cell, cystine is reduced to cysteine, which serves as the rate-limiting precursor for glutathione (GSH) biosynthesis. This process proceeds through sequential enzymatic steps catalyzed by glutamate–cysteine ligase (GCL) and glutathione synthetase (GSS). Acting as a critical reducing cofactor, GSH enables glutathione peroxidase 4 (GPX4) to convert lipid hydroperoxides into their corresponding lipid alcohols, thereby preventing lethal lipid peroxidation and maintaining redox homeostasis. The FSP1–CoQ_10_ and GCH1–BH4 systems can also inhibit ferroptosis. CoA-PUFAs, coenzyme A-polyunsaturated fatty acids; PL-PUFAs-OOH, phospholipid-bound polyunsaturated fatty acid hydroperoxides; PROM2, prominin 2; GSSG, glutathione disulfide; FSP1, ferroptosis suppressor protein 1; CoQ_10_, coenzyme Q10; GTP, guanosine triphosphate; GCH1, GTP cyclohydrolase 1; BH4, tetrahydrobiopterin; TCA, tricarboxylic acid cycle; VDACs, voltage-dependent anion channels; CoQ_10_H_2_, ubiquinol.

Ferroptosis is associated with the onset and advancement of PD. Its core pathophysiological characteristics are intricately intertwined with abnormal iron and lipid metabolism, oxidative stress, mitochondrial dysfunction, and neuroinflammation, driving the progressive degeneration of dopaminergic neurons in the substantia nigra (SN) (**Figure 2**).

### 2.1 Iron dysmetabolism in PD

Ferroptosis is fundamentally driven by dysregulated intracellular iron homeostasis, leading to iron-catalyzed lipid peroxidation (21, 31). A crucial initial event is the accumulation of the labile iron pool (LIP), predominantly consisting of catalytic ferrous iron (Fe^2+^). Through the Fenton reaction, Fe²^+^ facilitates the conversion of hydrogen peroxide into highly reactive hydroxyl radicals and other ROS (32). These radicals then attack the polyunsaturated fatty acid (PUFA) chains abundant in cellular membranes, initiating an uncontrolled chain reaction of lipid peroxidation that ultimately disrupts membrane structural and functional integrity, leading to cell death (33, 34).

#### 2.1.1 Regional iron accumulation

Significant alterations in iron distribution and accumulation occur in PD patient brains, a fact confirmed by postmortem studies and advanced neuroimaging techniques. Dexter et al. (1987) first reported nigral iron elevation in PD, a finding later confirmed by X-ray microanalysis (35, 36). A recent integrative study combining magnetic resonance imaging (MRI), quantitative susceptibility mapping (QSM), and regional gene expression profiling has revealed significantly elevated cortical iron accumulation in PD (37). Importantly, iron levels in the SNpc are associated with both motor disability and disease progression in PD (38).

#### 2.1.2 Dysregulation of key iron-regulating proteins

In ferroptosis, intracellular iron homeostasis is maintained by a network of regulatory mechanisms, including iron acquisition via the transferrin (Tf)/transferrin receptor 1 (TfR1) pathway, intracellular transport by divalent metal transporter 1 (DMT1), export by ferroportin (FPN1), storage in ferritin, and iron release through ferritinophagy mediated by nuclear receptor coactivator 4 (NCOA4) (39, 40).

Iron acquisition typically begins with extracellular ferric iron (Fe^3+^) binding to Tf, forming a Tf-Fe^3+^ complex that is internalized through TfR1-mediated endocytosis (41, 42). Mastroberardino et al. first revealed that the Tf/TfR2 pathway can directly import iron-loaded Tf into the mitochondria of dopaminergic neurons, and in the rotenone-induced PD model, this leads to mitochondrial iron deposition and excessive production of free radicals (43). Transcription factor EB (TFEB), a pivotal regulator of the autophagy-lysosome pathway, has been shown to promote the clearance of misfolded α-syn and prevent both ferroptosis and iron overload when overexpressed. In addition, elevated TFEB expression increases the production of TfR1 and drives its accumulation within lysosomes, which in turn facilitates iron uptake into lysosomal compartments and enables transient storage (44).

Inside the endosome, Fe^3+^ is reduced to Fe^2+^ by ferrireductases like six-transmembrane epithelial antigen of prostate 3 (STEAP3), and the resulting Fe^2+^ is then transported into the cytoplasm primarily by DMT1 (39, 45). Within the SN of PD patients, the expression of a specific subtype of DMT1 (+IRE) is significantly upregulated, accompanied by iron accumulation in dopaminergic neurons, enhanced oxidative stress, and cell death (46). In the 1-methyl-4-phenyl-1,2,3,6-tetrahydropyridine (MPTP) mouse model, DMT1 (+IRE) levels in the ventral mesencephalon are significantly upregulated at 1 and 2 days post-MPTP treatment (P < 0.01), and the total iron content in the ventral mesencephalon is correspondingly increased to 77.4 ± 4.2 ng/mg protein (vs. 59.8 ± 4.0 ng/mg protein in controls, P < 0.01) at 2 days post-treatment, indicating a positive temporal and spatial association between DMT1 (+IRE) upregulation and iron accumulation. Mice carrying a functionally defective DMT1 mutation exhibit marked resistance to MPTP-induced neurotoxicity, with only a 25% loss of SNpc tyrosine hydroxylase-positive (TH^+^) dopaminergic neurons compared with 53% and 58% loss in wild-type (+/+) and heterozygous (+/mk) mice, respectively. Similarly, rats harboring the same DMT1 G185R mutation show reduced susceptibility to 6-hydroxydopamine (6-OHDA)-induced neurotoxicity, with 42.6% dopaminergic neuron loss versus 61.6% in wild-type and 65.9% in heterozygous animals (46). This neuroprotective phenotype occurs despite comparable total brain iron levels and reflects impaired intracellular iron trafficking rather than altered total iron content.

Lysosomes serve as dynamic iron reservoirs and key regulators of cellular iron homeostasis. Under physiological conditions, ferritin-bound or excess Fe^2+^ is delivered to lysosomes via NCOA4-mediated ferritinophagy or through direct uptake by transporters such as ZIP8, ZIP14, and DMT1. Within lysosomes, iron is normally reduced and recycled back to the cytoplasm via TRPML1 or DMT1 to meet metabolic demands (47). However, in DMT1-mutant models, this recycling pathway is disrupted, leading to iron sequestration in lysosomes in a redox-inactive form, thereby lowering cytosolic Fe^2+^ levels and suppressing Fenton reaction–driven oxidative stress and ferroptosis.

The cytosolic and mitochondrial LIP can be utilized in enzymatic reactions or stored in ferritin, the major iron-storage protein composed of heavy (FTH1) and light (FTL) chain subunits. However, under certain conditions, ferritin is degraded through a selective autophagy process known as ferritinophagy, which is mediated by NCOA4. This process releases stored iron into the LIP, thereby increasing the cell’s susceptibility to ferroptosis (48, 49). In PD, exposure to paraquat (PQ) exemplifies this process, as PQ upregulates NCOA4 expression and consequently activates ferritinophagy, leading to degradation of ferritin and release of stored Fe^2+^ into the LIP. The resultant accumulation of labile iron intensifies oxidative stress via Fenton chemistry and ultimately renders dopaminergic neurons highly susceptible to ferroptosis (50).

Cells export iron via the plasma membrane iron exporter protein, FPN1, the sole identified protein responsible for exporting intracellular iron in mammals (51). The liver-produced hormone hepcidin induces its internalization and degradation, while overexpression of SLC40A1 inhibits ferroptosis (52). Hepcidin knockdown by siRNA increases the viability of 6-OHDA-treated N27 dopaminergic neuronal cells by 22%. This protective effect is achieved through the upregulation of FPN1 expression, which reduces intracellular iron content, attenuates ROS production, and inhibits apoptosis (evidenced by reduced caspase-3 activation and DNA fragmentation) (53). These results indicate that enhancing FPN1-mediated iron efflux can inhibit iron-dependent lipid peroxidation.

Ceruloplasmin (CP) is a ferroxidase that is mainly found in a GPI-anchored form on astrocytes, converting toxic Fe^2+^ to non-toxic Fe^3+^, physically associating with FPN1 to facilitate iron efflux from central nervous system cells, and inhibiting Fe^2+^-mediated lipid peroxidation by reducing the presence of highly reactive Fe^2+^ (54, 55). In PD patients, nearly 80% loss of CP ferroxidase activity in the SN may lead to pro-oxidative iron accumulation, as evidenced by CP knockout mice developing parkinsonian symptoms reversible by iron chelation (56). Analysis of cerebrospinal fluid (CSF) from PD patients by Olivieri et al. revealed increased charge heterogeneity of CP, indicating oxidative modification, accompanied by a significant decrease in its ferroxidase activity. *In vitro* experiments demonstrated that functionally impaired CP fails to effectively oxidize Fe^2+^, leading to iron ion retention in neuronal cells and promotion of ferroptosis (57).

Iron regulatory proteins (IRPs), bind to iron-responsive elements (IREs) in the 5’ untranslated region (UTR) of mRNAs encoding ferritin and FPN1 to inhibit their translation, while binding to IREs in the 3’ UTR of TfR1 and DMT1 mRNAs to stabilize these transcripts, thereby adjusting iron storage, export, and uptake (41, 58). A study found that the expression of IRP2 is elevated in MPTP-induced PD mouse models. Mice with IRP2 overexpression exhibit motor dysfunction along with increased TfR1, enhanced iron deposition, and upregulation of ferroptosis markers in the SN. This suggests that elevated IRP2 leads to iron metabolism disorders and promotes neurodegeneration. Further studies indicate that IRP2 binds to p53 and activates the SLC7A11-ALOX12 pathway, thereby inducing ferroptosis (59).

#### 2.1.3 The dual role of NM in iron homeostasis and PD pathology

NM-containing neurons in the SNpc and the locus coeruleus are among the most vulnerable and prominently degenerated cell types in PD. The expression level of ferritin is relatively low in SN dopaminergic neurons, while NM serves as their primary iron storage system. In healthy individuals, the NM content in the SN begins to accumulate at approximately 3 years of age and continues to increase with age, reaching concentrations of 2–4 mg per gram of wet tissue after the age of 50 (60). NM is a complex macromolecule mainly composed of melanin components such as eumelanin and pheomelanin (61). Research indicates that NM exerts dual neuroprotective and neurotoxic effects.

Physiologically, NM acts as a neuroprotective agent. The synthesis of NM stems from the non-enzymatic auto-oxidation of dopamine, a process that can eliminate excess dopamine and its toxic intermediate products (e.g., dopaquinone), preventing the accumulation of cytotoxic substances (62, 63). Meanwhile, nigral NM can chelate iron ions through high-affinity binding sites, reducing iron-mediated oxidative stress (such as the Fenton reaction) and exerting antioxidant protective effects (60, 63).

However, under pathological conditions in PD, NM can manifest neurotoxic properties. A recent study identified increased levels of pro-oxidant pheomelanin and decreased levels of eumelanin in nigral NM of PD patients (64). Moreover, neuronal death is promoted by synthetic pheomelanin *in vitro*, whereas synthetic eumelanin exhibits no neurotoxic effects. This suggests a shift towards a more pro-oxidant form of NM in PD.

Extracellular NM released by dying dopaminergic neurons plays a critical role in triggering chronic neuroinflammation in PD. In rodent models overexpressing human tyrosinase, the extracellular NM activates microglia, leading to their phagocytosis and degradation of the pigment, which indicates an active neurodegenerative process (65). Furthermore, NM-rich neurons exhibit accumulation of immunoglobulin G and expression of major histocompatibility complex class I antigens, directly correlated with neuronal loss. Concurrently, serum levels of anti-melanin antibodies are markedly elevated in PD patients. The positive rate reaches 46.7% (28/60 cases), significantly higher than 13.3% (8/60 cases) in healthy controls (p < 0.001). Consistently, the mean ELISA absorbance value reflecting antibody concentration is also higher in PD patients (0.38 ± 0.12) than in controls (0.15 ± 0.08) (p < 0.001) (65, 66).

Evidence also shows that the progressive build-up of NM over time in SN dopaminergic neurons may induce abnormal misfolding of α-syn (67). Importantly, this synucleinopathy can then spread anterogradely to the cerebral cortex, further implicating NM in the broader pathogenesis of PD.

### 2.2 Lipid peroxidation in PD

Abnormal lipid metabolism and subsequent lipid peroxidation are pivotal pathological features commonly observed in PD and ferroptosis. Lipids are most abundant in the human brain among all tissues except adipose tissue (68). PUFAs, due to the inherent instability of their bis-allylic hydrogen atoms, are highly vulnerable to oxidative damage and act as the primary substrates for ferroptotic signaling (69). Altering the PUFA composition of dopaminergic neuronal membranes and co-treating with iron can synergistically amplify lipid peroxidation and trigger ferroptosis, which has been confirmed in LUHMES cell and mouse organotypic brain slice experiments (70).

Enzymes like acyl-CoA synthetase long-chain family member 4 (ACSL4) and lysophosphatidylcholine acyltransferase 3 (LPCAT3) are central to incorporating PUFAs into membranes, sensitizing cells to ferroptosis (71–73). Consequently, inhibition of ACSL4 or LPCAT3 reduces these oxidizable phospholipid-polyunsaturated fatty acids (PL-PUFAs), rendering cells resistant to ferroptosis (74, 75). β-hydroxybutyric acid, a type of ketone body, has been found to have neuroprotective effects. β-hydroxybutyric acid upregulates the expression of zinc finger protein 36, which facilitates the binding and degradation of ACSL4 mRNA, thereby reducing ACSL4 levels. This process subsequently suppresses oxidative stress and ferroptosis, ultimately protecting dopaminergic neurons from injury (76).

As non-heme iron-containing dioxygenases, lipoxygenases (LOXs) directly catalyze the peroxidation of PUFAs, both free and esterified in phospholipids (77, 78). Specifically, 15-LOX selectively catalyzes the oxidation of phosphatidylethanolamine-arachidonic acid, generating 15-hydroperoxy-eicosa-tetra-enoyl-phosphatidylethanolamine (15-HpETE-PE), which serves as a signal for ferroptosis (79, 80). RSL3-treated PD patient-derived fibroblasts exhibited a significant increase in both 5-hydroxyeicosatetraenoic acid and 15-hydroxyeicosatetraenoic acid levels in the conditioned medium, as revealed by lipidomic analysis (81).

PTC-041, a selective 15-LOX inhibitor, blocks lipid peroxidation and the ferroptosis pathway by inhibiting enzyme activity in its reduced hydroquinone form. Recent research indicates that PTC-041 inhibits ferroptosis, reduces lipid oxidation, and enhances cell viability in fibroblasts derived from PD patients (81). Additionally, PTC-041 inhibits the aggregation, nitrosylation, and phosphorylation of α-syn in N27 dopaminergic cells, thereby reducing cytotoxicity. In LUHMES cell models of PD, RSL3 induces ferroptosis, while ACSL4 inhibitors (such as troglitazone), ALOX15/15B inhibitors (such as PD 146176, baicalein), and siRNAs targeting ACSL4, ALOX15, and ALOX15B all effectively inhibit ferroptosis and protect cells (70).

Lipid peroxidation not only directly damages cell membrane phospholipid structure and neuronal function but also decomposes into highly reactive electrophilic aldehydes including 4-hydroxynonenal (4-HNE) and malondialdehyde (MDA) (82, 83). These byproducts can covalently modify proteins, DNA, and other lipids, exacerbating cellular damage and contributing to the ferroptosis (84, 85).

Elevated levels of HNE-protein adducts have been observed both in Lewy bodies within the SN and in the CSF of PD patients (82, 86). Early compensatory changes in dopamine turnover, stemming from nigral cell degeneration, were shown to exacerbate oxidative stress and promote 4-HNE accumulation in the SN (87). 4-HNE is involved in PD pathology by covalently modifying α-syn to promote its aggregation, disrupting the ubiquitin-proteasome system, and interfering with dopamine receptor function, and has been found to alter dopamine transport, contributing to dopamine loss (82, 86, 88). Clearing 4-HNE or enhancing its detoxification capacity through acetaldehyde dehydrogenase and GSH pathways has shown neuroprotective effects in PD models (88).

Increased concentrations of MDA have been detected in PD patients at both early and advanced stages (201.9 ± 18.9 μM/mg protein and 214.1 ± 29.7 μM/mg protein, respectively), significantly higher than in healthy controls (168.1 ± 18.3 μM/mg protein; p < 0.001), suggesting its potential as a disease biomarker (89). A very recent study demonstrates that MDA covalently modifies α-syn, altering its structure and enhancing its aggregation propensity. The formed MDA-modified α-syn induces irreversible motor dysfunction, causes SN dopaminergic neuronal loss, promotes protein aggregation, and leads to other PD-like pathological features in mice. Moreover, MDA promotes the aberrant aggregation of α-syn by modifying its 15 lysine sites, thereby initiating Lewy body formation and driving the pathological process of PD (90).

### 2.3 Oxidative stress and dysregulation of antioxidant systems in PD

Oxidative stress is critically involved in PD pathophysiology. One of the major antioxidant pathways counteracting ferroptosis is System Xc^-^-GSH-GPX4. System Xc^-^, a cystine/glutamate antiporter, functions as a transmembrane complex of light chain xCT (*SLC7A11*-encoded) and heavy chain 4F2hc (72). Hypermethylation at the cg06690548 locus in the *SLC7A11* promoter correlates with higher PD susceptibility (91). This epigenetic modification suppresses *SLC7A11* transcription, thereby reducing xCT expression, impairing cystine uptake, and limiting intracellular GSH synthesis, ultimately heightening neuronal vulnerability to ferroptosis (92). This series of changes is highly consistent with pathological changes such as reduced GSH and increased oxidative stress in the SN of PD patients. Indeed, GSH levels are markedly decreased in the SN of PD patients, which exacerbates oxidative injury and promotes ferroptosis (93). Post-mortem analyses have demonstrated a significant reduction of GPX4 in the SN of PD brains (94). However, other studies report elevated GPX4 expression in PD patients, possibly reflecting a compensatory response (95).

Ferroptosis suppressor protein 1 (FSP1), originally identified as apoptosis-inducing factor mitochondria-associated 2 (AIFM2), was rediscovered through unbiased genetic screens as a potent GPX4-independent ferroptosis inhibitor (96). Mechanistically, FSP1 functions at the membrane as an NAD(P)H-dependent CoQ_10_ oxidoreductase, reducing CoQ_10_ to its active form, CoQ_10_H_2_, which is a potent antioxidant for mitochondria and lipids (97–99).

PD patients and animal models exhibit decreased CoQ_10_ levels (100, 101). In various mammalian PD models, the elevated expression of NADPH oxidase 1 (Nox1) and NADPH oxidase 4 (Nox4) is a key event driving oxidative damage to dopaminergic neurons and disease progression. Nox1 forms an intranuclear complex with Ras-related C3 botulinum toxin substrate 1, directly mediating oxidative DNA damage and activation of apoptotic pathways. The expression changes of Nox1 are closely associated with neurotoxin-induced acute injury (102). Nox4 participates in the chronic progression of PD through the synergistic effect of intranuclear ROS generation and α-syn pathological toxicity (103).

An additional antioxidant mechanism implicated in ferroptosis is the GTP cyclohydrolase-1 (GCH1)-tetrahydrobiopterin (BH4)-phospholipid axis. Beyond direct radical trapping, elevated BH4 levels can also promote the synthesis of CoQ_10_ (104), further enhancing ferroptosis resistance. As a cofactor of tyrosine hydroxylase (TH), BH4 is involved as TH catalyzes dopamine synthesis. In PD patients, reduced BH4 levels and GCH1 activity in the SN and striatum are associated with PD progression, indicating that aberrant BH4 metabolism contributes to PD pathogenesis (105). Moreover, GCH1 has been identified as a genetic risk factor for PD (106).

### 2.4 Mitochondrial dysfunction in PD

Mitochondrial dysfunction is intricately linked to PD etiology and ferroptosis. Ferroptotic cells exhibit distinctive mitochondrial morphology, characterized by shrinkage, increased electron density, reduced cristae, and outer membrane rupture compared to normal cells (21, 107, 108). Mitochondrial dysfunction elicits elevated ROS levels, which in turn promotes lipid hydroperoxide accumulation, potentially spreading beyond the mitochondrial compartment (109, 110). Mitochondria are also crucial for cellular iron metabolism, importing iron via outer membrane voltage-dependent anion channels (VDACs) and inner membrane Mfrn1/2, though mitochondrial iron exporters like CISD1/2 also exist (111, 112). The mitochondrial electron transfer chain (ETC) exerts a dual role in ferroptosis. While ETC activity drives pro-ferroptotic ROS generation via electron leakage, it is also essential for anti-ferroptotic enzymes (e.g., DHODH and GPD2) that reduce mitochondrial coenzyme Q (CoQ) to ubiquinol (CoQH_2_) (113–115).

Mitochondrial damage can trigger mitochondrial DNA (mtDNA) release, activating the cGAS-STING1 pathway and potentially contributing to ferroptosis, as observed with inducers like zalcitabine (116). MtDNA is particularly vulnerable to damage. In early-stage PD patients, somatic mtDNA point mutations are markedly elevated in SN dopaminergic neurons, with respiratory chain defects resulting from mtDNA deletions being particularly prominent (117, 118).

Mitochondrial toxins including MPTP and rotenone can induce PD-like symptoms, confirming a direct association between mitochondrial function and PD (119). Iron-driven oxidation of dopamine results in the generation of 6-OHDA, which specifically inhibits mitochondrial complexes I and IV, leading to mitochondrial dysfunction (120). In idiopathic PD patients, dopaminergic neurons in the SNpc exhibit significant complex I deficiency, characterized by a reduced proportion of complex I-positive neurons, whereas functional abnormalities of other complexes are not significant (121).

The mitochondrial division inhibitor−1 (Mdivi-1) protein, a dynamin-related protein 1 inhibitor, can inhibit mitochondrial fission, reduce neuronal death and protect neurons. Studies found that Mdivi-1 rescued evoked dopamine release in *PINK1* knockout mice and improved evoked dopamine release in mice pre-lesioned with MPTP (122). In the A53T-α-syn rat model, intraperitoneal injection of Mdivi-1 protected against nigrostriatal degeneration, as shown by more dopaminergic neurons in the SN compared to untreated animals (123).

## 3 The pathological links and genetic intersections in PD and ferroptosis

The onset and development of PD are closely associated with impairments in mitochondrial function. These disturbances, often influenced by intricate genetic determinants, can trigger multiple forms of programmed death in dopaminergic neurons, among which ferroptosis plays a significant role (119, 124).

### 3.1 PD pathological features induce ferroptosis susceptibility

#### 3.1.1 SNpc dopaminergic neuron death and ferroptosis

A defining pathological feature of PD is the selective death of dopaminergic neurons within the SNpc (125). Dopaminergic neurons are especially prone to ferroptotic damage, partly because dopamine metabolism itself can generate reactive quinone species, contributing to oxidative stress. Under physiological pH, Fe^3+^ interacts with dopamine to form a binding complex, facilitating dopamine entry into dopaminergic neurons. When the cellular environment is under oxidative stress, Fe^3+^ acts as a catalyst to drive the non-enzymatic oxidation of dopamine, leading to the formation of dopamine-quinone derivatives that can bind iron to form iron-dopamine-quinone complexes. These unstable intermediate complexes can undergo further chemical transformations into toxic dopamine-derived species, and such dopamine-quinone derivatives are well-documented to impair mitochondrial function and exert direct neurotoxic effects on SN dopaminergic neurons, contributing to their selective vulnerability in pathological states like PD (126).

The metabolic pathway for dopamine synthesis in dopaminergic neurons involves TH and is critically supported by iron as a synergistic cofactor. Intriguingly, Dichtl et al. identified that, uniquely among dopamine precursors or metabolic components, dopamine itself induces cellular iron accumulation during its metabolism (127). This phenomenon, proposed to occur through enhanced uptake of non-ferritin-bound iron and decreased iron elimination, leads to higher cellular iron concentrations. These findings highlight a pivotal regulatory role for dopamine in iron deposition, which may be directly relevant to the iron overload and subsequent ferroptosis in SNpc dopaminergic neurons.

Dopamine metabolism is also linked to NM. Under physiological conditions, NM synthesis from dopamine autooxidation is neuroprotective, chelating iron and preventing cytotoxic substance accumulation. However, in pathological states, NM can become neurotoxic. Elevated intracellular iron can saturate NM’s binding sites, causing iron release from low-affinity sites and enhancing iron-driven oxidative stress (128).

Dopamine itself can act as a strong inhibitor of ferroptosis. A study demonstrated in four cell lines—PANC1, HEY, MEF, and HEK293—that non-oxidized dopamine dose-dependently (12.5–50 μM) inhibited GPX4 protein degradation induced by 20 μM erastin. This preservation of GPX4 maintained its ability to reduce lipid hydroperoxides using GSH as a co-substrate. Concurrently, non-oxidized dopamine lowered intracellular Fe^2+^ accumulation by approximately 30–40% at 25 μM, decreased MDA production by about 25–35% at 25 μM, and sustained GSH levels restored by roughly 20–30% compared with the erastin group. As a result, cell viability increased from about 40% in the erastin-treated group to nearly 80% at 50 μM dopamine, thereby effectively suppressing ferroptosis (129). Consequently, the decreased dopamine levels characteristic of PD may render SNpc neurons more vulnerable to ferroptosis.

#### 3.1.2 α-syn aggregation induces ferroptosis

The aggregation of α-syn, a core pathological feature of PD (130), can directly induce ferroptosis. Oligomeric α-syn interacts with cellular membranes to accelerate lipid peroxidation and integrates into the cytomembrane, leading to calcium influx, elevated intracellular calcium levels, and subsequent excitotoxicity and ferroptotic cell death (25).

The process through which α-syn oligomeric species trigger the generation of ROS and the peroxidation of lipids also exhibits a reliance on iron. Fe^3+^ demonstrates a stronger binding affinity for α-syn (131), potentially impacting α-syn post-translational modifications or influencing Fe^2+^ oxidation, thereby altering α-syn fibril morphology and accelerating its aggregation (132). Intraneuronal aggregates of α-syn that are intracellularly localized and bound to cellular membranes are capable of triggering ferroptosis through these aggregate-membrane interactions (25). Collectively, these findings indicate a bidirectional enhancement of α-syn aggregation and ferroptosis, driving dopaminergic neuron deterioration.

### 3.2 Relationship between PD-related genes/proteins and ferroptosis

PD pathophysiology is significantly influenced by genetic predisposition, with numerous gene loci linked to its development (133). These genes, encompassing both risk-associated genes for sporadic PD (e.g., *SNCA*, *LRRK2*, *GBA*) and those associated with familial forms (e.g., *PARK2*, *PINK1*, *PARK7*), predominantly encode proteins involved in critical cellular functions like oxidative stress response, mitochondrial quality control, and protein degradation pathways.

**Figure 3:**
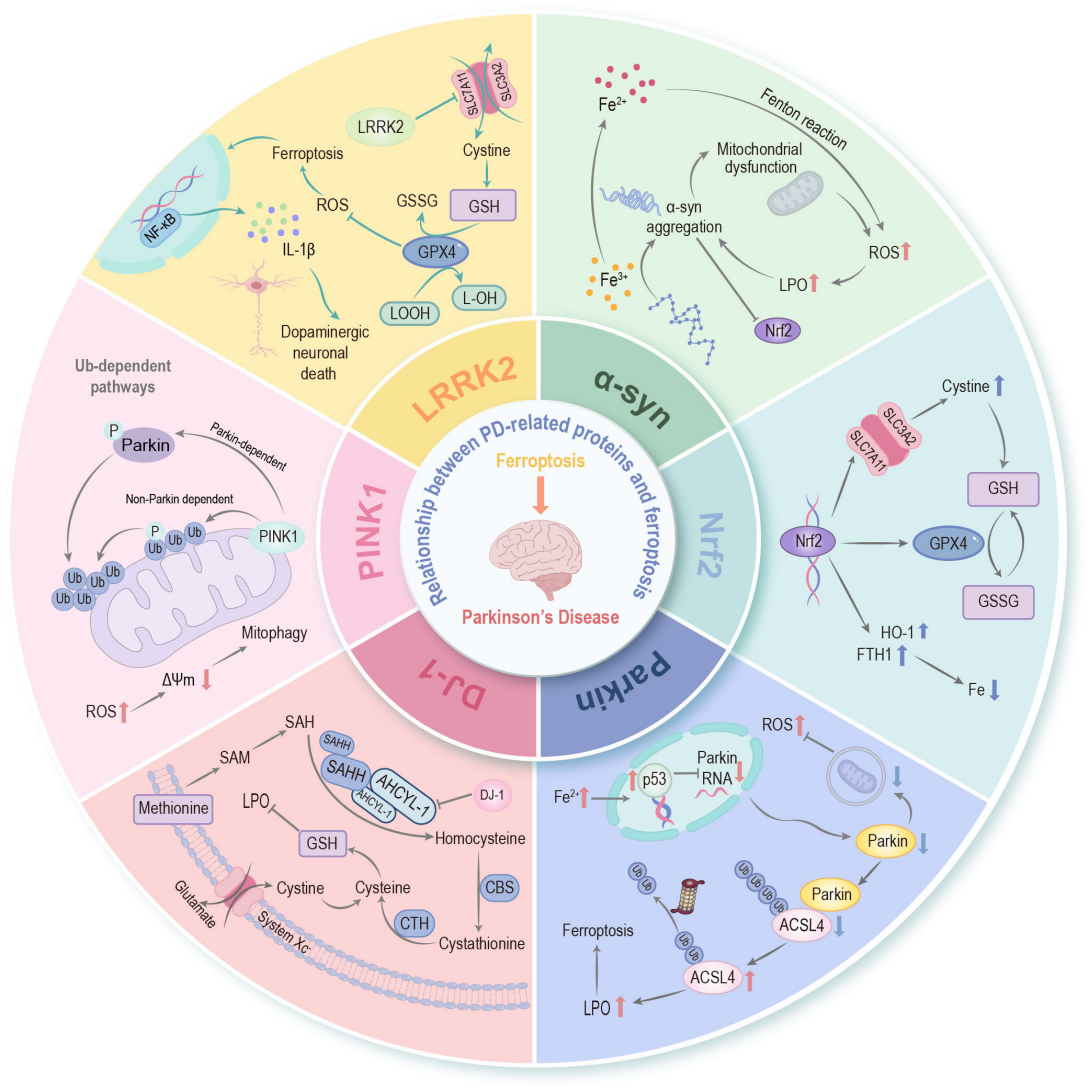
Relationship between PD-related proteins and ferroptosis. An association exists between ferroptosis and PD-related proteins—including α-synuclein, Parkin, PINK1, DJ-1, LRRK2, etc.—as well as with Nrf2, which serves as the “master regulator” of cellular redox homeostasis. These PD-associated proteins establish intricate crosstalk with ferroptosis through the regulation of iron homeostasis, lipid peroxidation cascades, mitochondrial bioenergetics, and the glutathione-dependent antioxidant network, thereby synergistically facilitating the pathological progression of PD. α-syn, α-synuclein; PINK1, PTEN induced putative kinase 1; LRRK2, leucine-rich repeat kinase 2; Nrf2, nuclear factor erythroid 2-related factor 2; ROS, reactive oxygen species; LPO, lipid peroxidation; p53, tumor protein p53; ACSL4, long-chain acyl-CoA synthetase 4; SAM, S-adenosylmethionine; SAH, S-adenosyl-L-homocysteine; SAHH, S-adenosylhomocysteine hydrolase; AHCYL1, adenosylhomocysteinase like protein 1; CBS, cystathionine-β-synthase; CTH, cystathionine gamma-lyase; Ub, ubiquitin; Δψm, mitochondrial membrane potential; NF-κB, nuclear factor-kappa B; GPX4, glutathione peroxidase 4; GSH, glutathione; SLC7A11, solute carrier family 7 member 11; LOOH, lipid hydroperoxide; L-OH, lipid alcohol; GSSG, glutathione disulfide; IL-1β, interleukin-1 beta; HO-1, heme oxygenase-1; FTH1, ferritin heavy chain 1.

Altered biochemical functions of these protein molecules can profoundly modulate cellular sensitivity to ferroptosis. For instance, the intricate link between PD etiology and mitochondrial dysfunction, frequently modulated by complex genetic factors (124), may impact ferroptosis susceptibility. Given that many PD-associated proteins regulate mitochondrial health, oxidative stress, and iron homeostasis, their dysregulation provides a direct mechanistic bridge to ferroptosis in PD progression (**Figure 3**).

#### 3.2.1 α-syn

As key neuropathological features of PD, α-syn aggregation and iron deposition exhibit a profound interplay (134). Iron can directly promote α-syn aggregation through structural links, leading to conformational changes (135). Conversely, α-syn perturbs iron homeostasis via its ferrireductase activity, leading to elevated intracellular Fe^2+^ levels (136). Furthermore, α-syn exacerbates mitochondrial dysfunction by binding to VDAC, where it partially obstructs the pore and thereby impairs the transport of ATP/ADP and other metabolites. Meanwhile, α-syn can also be transported into mitochondria via VDAC, interfering with mitochondrial respiration (137).

α-syn is increasingly shown to be involved in regulating lipid metabolism (10). The N-terminal domain of α-syn preferentially binds to negatively charged lipids, influencing membrane lipid composition and fluidity (138). This binding can induce conformational changes in α-helices, which in turn regulates lipid homeostasis. For instance, overexpression of α-syn increases the levels of monounsaturated fatty acids, altering the membrane lipid balance and exacerbating toxicity (139). Conversely, dysregulation of lipid metabolism triggers the aggregation of α-syn, forming a vicious cycle (138).

Direct evidence from human dopaminergic neuronal models indicates that reducing α-syn accumulation can prevent ferroptosis (25, 140). Mutations in the *SNCA* gene promote α-syn aggregation into oligomers or fibrils, which disrupt the ubiquitin-proteasome system and impair membrane binding by reducing its N-terminal α-helical structure, leading to compromised membrane integrity and increased permeability (141, 142).

#### 3.2.2 Parkin (*PARK2*)

Parkin, encoded by *PARK2*, is an E3 ubiquitin-protein ligase critical in protein homeostasis via monoubiquitylation, multiple monoubiquitylation, and K48-/K63-linked polyubiquitylation. It regulates mitochondrial dynamics and protects dopaminergic neurons. Autosomal recessive *PARK2* mutations, a leading cause of early-onset PD, reduce or eliminate Parkin, impairing mitophagy, causing damaged mitochondria to accumulate, increasing oxidative stress, and triggering dopaminergic neuron death, thus driving PD progression (143).

Parkin is critical for mitochondrial quality control, mainly through mitophagy to clear damaged mitochondria and thereby effectively reducing the accumulation of ubiquitin-dependent misfolded proteins (144, 145). Given that mitochondrial dysfunction is an important factor for ferroptosis (146), Parkin’s role in mitophagy suggests a potential link.

The VDACs located on the outer mitochondrial membrane are key convergence points. When mitochondrial function is disrupted, they specifically interact with Parkin, acting as anchoring platforms for the recruitment of cytosolic Parkin to damaged mitochondria. This recruitment is crucial for efficient mitophagy: depleting all three VDACs impairs Parkin’s targeting to defective mitochondria and mitochondrial degradation, yet each individual VDAC can redundantly support Parkin recruitment and mitophagy (147).

More direct studies indicate that VDAC2/VDAC3 are early targets of erastin-induced ferroptosis, and both the opening of VDAC and carbonylation are strongly linked to the initiation of ferroptosis (148). Furthermore, in other disease models (such as Aβ-induced nerve cell damage), highly expressed or activated VDAC1 has been clearly involved in ferroptosis, exacerbating cell death by inhibiting anti-ferroptosis pathways (such as GPX4, AMPK/mTOR, Wnt/β-catenin) (149).

Further linking Parkin to ferroptosis, studies in cardiac pathology have revealed its crucial protective role. Normally highly expressed in the heart, Parkin degrades ACSL4 via ubiquitination, thereby inhibiting the production of PL-PUFAs and suppressing ferroptosis in cardiomyocytes. However, during myocardial infarction, increased iron ions activate p53, which transcriptionally inhibits Parkin expression, consequently promoting myocardial ferroptosis and exacerbating infarction (150).

Moreover, Parkin plays a role in regulating microglial and astrocyte activation (151, 152), processes that are deeply intertwined with neuroinflammation and oxidative stress, both of which can influence ferroptosis susceptibility in PD. Parkin deficiency may affect the microglial inflammatory activation by enhancing NF-κB-dependent NLRP3 activation (153). In Parkin-knockout BV2 microglial cells, necroptosis is reduced, which may prolong the inflammatory state (154). Astrocytes with Parkin deficiency exhibit enhanced endoplasmic reticulum stress, elevated cytokine release, and reduced neurotrophic factor secretion under stress, rendering neurons more susceptible to neurotoxins (155).

#### 3.2.3 PINK1

PINK1 is a serine/threonine protein kinase localized in mitochondria. When mitochondrial membrane potential is lost, it cannot be normally imported into the inner membrane. Instead, it accumulates on the outer mitochondrial membrane and binds to the translocase of the outer mitochondrial membrane complex. The accumulation of PINK1 on damaged mitochondria recruits Parkin and activates its activity. Once activated, Parkin mediates the ubiquitination of outer mitochondrial membrane proteins such as Mfn1/2 and Miro, and recruits the autophagy receptor p62, thereby initiating the autophagic degradation (mitophagy) of damaged mitochondria to maintain mitochondrial function (156). Like Parkin, PINK1’s involvement in mitochondrial quality control suggests a potential influence on ferroptosis.

#### 3.2.4 DJ-1 (*PARK7*)

DJ-1 is a multifunctional protein that inhibits oxidative damage by promoting GSH synthesis and maintaining mitochondrial function (3, 157). E64D mutation of DJ-1 disrupts mitochondrial respiration and enhances ROS production (158). Direct evidence of DJ-1’s role in neuronal survival against ferroptosis comes from studies showing that dopaminergic neurons differentiated from induced pluripotent stem cells of *PARK7* mutant carriers exhibit increased sensitivity to ferroptosis (93). Mechanistically, DJ-1 can negatively regulate ferroptosis by maintaining the biosynthesis of cysteine and GSH. Furthermore, the *SLC7A11* gene, which is often significantly downregulated in PD, is also considered a critical regulator of ferroptosis and can protect dopaminergic neurons by activating the transsulfuration pathway, indicating a potential interplay with DJ-1 (93).

DJ-1 functions as a bona fide negative modulator of ferroptosis by sustaining S-adenosyl homocysteine hydrolase activity through altering its interaction with the negative regulator adenosylhomocysteinase like protein 1, thereby maintaining the transsulfuration pathway-mediated biosynthesis of homocysteine and subsequent GSH synthesis (157). Beyond its direct cellular roles, DJ-1 is also involved in modulating astrocyte activation (151, 159).

#### 3.2.5 LRRK2

The leucine-rich repeat kinase 2 (LRRK2) participates in mitochondrial dynamics, oxidative stress, and lipid metabolism (160). *LRRK2* mutations (such as G2019S) promote mitochondrial fission in microglia by enhancing kinase activity, releasing mtROS and other factors to activate the NLRP3 inflammasome, exacerbating neuroinflammation in PD, and their regulation of mitochondrial dynamics has potential associations with ferroptosis (124). In MPTP-treated mice, LRRK2 inhibition diminishes microglial activation, protects dopaminergic neurons from apoptosis, and upregulates the p62-Keap1-Nrf2 pathway (161).

Additionally, abnormal activation of LRRK2 downregulates SLC7A11 in microglia, impairing cystine import to inhibit GSH synthesis. Reduced GSH decreases GPX4 expression/activity, promoting Fe^2+^ and ROS accumulation. This activates NF-κB to trigger neuroinflammation, ultimately exacerbating SNpc dopaminergic neuron apoptosis in PD (162). Moreover, iron itself can enhance LRRK2 activation. In the 6-OHDA-induced PD model, iron promotes LRRK2 activation via phosphorylation at S935 and S1292 sites. Activated LRRK2 then increases intracellular iron content by enhancing Fe^2+^ uptake, which in turn induces ROS generation and pro-apoptotic molecule expression. This forms an “iron-LRRK2” positive feedback loop that exacerbates dopaminergic neuron apoptosis (163).

#### 3.2.6 Nrf2 (*NFE2L2*)

Nuclear factor erythroid 2-related factor 2 (Nrf2) is a key regulator of cellular redox homeostasis (164, 165). The p62-Keap1-Nrf2 pathway can be activated by ferroptosis inducers, promoting the transcription of heme oxygenase-1 (*HMOX1*), *FTH1* and quinone oxidoreductase-1(*NQO1*) (164, 166). Nrf2 directly upregulates SLC7A11 expression, increases cystine uptake and subsequent GSH synthesis, thereby enhancing resistance to ferroptosis (167). Furthermore, Nrf2 regulates the expression of key GSH-dependent enzymes like GPX4, and other genes involved in GSH synthesis and regeneration (166, 168). Beyond GSH metabolism, Nrf2 also promotes intracellular NADPH generation by upregulating the expression of genes related to the pentose phosphate pathway, which contains the key enzyme G6PD, as well as genes involved in pyruvate cycling and folate metabolism (166). This comprehensive transcriptional regulation by Nrf2 impacts multiple nodes of ferroptosis, maintaining intracellular antioxidant balance and alleviating cellular injury, as evidenced in diverse *in vivo* and *in vitro* models (169).

In the SN neurons of PD, the expression of Nrf2 in the nucleus is enhanced, and the expression of its signature proteins NQO1 and HO-1 is upregulated, suggesting a brain protective effect mediated by Nrf2. In the PD *Drosophila* model, overexpression of Nrf2 and knockdown of Keap1 alleviate the decline in locomotor ability (170). In both 1-methyl-4-phenylpyridinium (MPP^+^)-exposed SH-SY5Y cells and MPTP-administered mice, Nrf2 activation regulates the upregulation of brain-derived neurotrophic factor (BDNF) transcription, thereby ameliorating dopaminergic neurotoxicity (171). These findings suggest that Nrf2 exerts a pivotal function in counteracting inherent oxidative stress throughout the pathological process of PD, thereby further supporting its therapeutic potential.

#### 3.2.7 Other PD-related genes/proteins potentially associated with ferroptosis

The absence of CISD1, an iron-rich outer mitochondrial membrane protein, triggers mitochondrial dysfunction alongside the depletion of striatal dopamine and TH, thus facilitating the advancement of PD and the worsening of its clinical manifestations (172). CISD1 is regulated by PINK1 and Parkin, two key players in mitochondrial quality control. It has been demonstrated that loss of PINK1 function leads to increased formation of CISD1 dimers, which are unable to coordinate iron-sulfur clusters and thus impair iron metabolism (173).

CHCHD2 stabilizes OPA1, a crucial protein for mitochondrial fusion (174), thereby contributing to mitochondrial integrity. *CHCHD2* mutations can trigger protein clumping in the intermembrane space of mitochondria (175), damaging the ETC, augmenting ROS, and leading to aberrant mitochondrial activity and dopaminergic neuron loss (176).

Other genes implicated in PD pathogenesis, such as *UCHL1, GBA, VPS35*, and *ATP13A2* (177, 178), also have established roles in influencing mitochondrial function and integrity. Their precise involvement in ferroptosis pathways warrants further investigation. Mutations in genes like *PLA2G6*, involved in phospholipid hydrolysis, are linked to neurodegenerative diseases with brain iron accumulation, sharing features with PD (179). Genes associated with lipid metabolism, such as synaptojanin1 (*SYNJ1*) (180, 181), are linked to PD risk and show altered lipid profiles or functions in models.

## 4 Ferroptosis in animal models of PD

Ferroptosis, along with its linked pathological features, has been detected in a range of PD animal models, indicating it could serve as a key mode of cell death in such model systems (27, 182). Research on these models, including neurotoxin-induced models, genetic models, and *in vitro* experiments involving iron overload and ferroptosis induction, helps to verify the role of ferroptosis in PD pathogenesis.

### 4.1 Neurotoxic models

In MPTP models, elevated levels of ACSL4 and elevated iron levels coexist with ferroptosis (183, 184). *In vitro* experiments have consistently demonstrated that 6-OHDA induces ferroptosis in SH-SY5Y neuroblastoma cell models. The p62-Keap1-Nrf2 pathway activation protects dopaminergic neurons from 6-OHDA-triggered ferroptosis (185). Lipopolysaccharide (LPS) model is suitable for studying the association between inflammation and PD. In a recent study, a PD-like rat model was successfully established via FeSO_4_-LPS co-exposure. It confirmed ferroptosis as the core mechanism in PD pathogenesis synergistically induced by iron overload and inflammation, which activate ferroptosis by disrupting iron metabolism, inhibiting the antioxidant system and exacerbating lipid peroxidation, further causing dopaminergic neuronal damage, α-syn aggregation, and PD-characteristic motor/cognitive impairments (186).Treating SH-SY5Y dopamine neurons with rotenone and using RhoNox-1 staining, followed by confocal microscopy observation and flow cytometry analysis, revealed significantly higher intracellular Fe^2+^ in the rotenone group, confirming cellular iron overload (187). Interestingly, when administered by gavage, iron carbonyl regulates iron absorption and utilization, maintains high aconitase 1 levels, increases GSH levels, reduces oxidative stress, and may prevent PD in rats subcutaneously injected with rotenone (188).

PQ generates free radicals through oxidative stress, which can induce dopaminergic neuron death and aggregation of α-syn (189). In both *in vitro* and *in vivo* models, PQ induces ferroptosis and iron overload by significantly increasing iron accumulation in the cytoplasm and mitochondria via the NCOA4-mediated ferritinophagy pathway. Additionally, PQ downregulates SLC7A11 and GPX4 expression and upregulates cyclooxygenase-2 expression, thereby promoting oxidative stress and ferroptotic cell death (50).

### 4.2 Genetic models

#### 4.2.1 *SNCA* models

Overexpression of wild-type or mutant (A53T, A30P) α-syn induces Lewy body-like inclusions in dopaminergic neurons, leading to significant neuron damage and degeneration. In α-syn^A53T/A53T^ mice exhibiting PD phenotypes, downregulation of GPX4 was observed in the SN at 3 months of age, while the ratio of Bcl-2/Bax (related to apoptosis) remained unchanged. It was not until 6 months of age that both ferroptosis and apoptosis characteristics appeared simultaneously, confirming that ferroptosis occurs earlier than apoptosis in PD pathogenesis (190).

As a chemokine receptor expressed on microglia surfaces, Cx3cr1 deficiency impairs normal microglial regulation. This deficiency makes microglia more susceptible to activation by α-syn^A53T^, thereby leading to excessive proliferation. Castro-Sánchez et al.’s study demonstrated that in *Cx3cr1*^−/−^ mice overexpressing α-syn^A53T^, neurodegeneration, neuroinflammation, and microglial activation were significantly enhanced (191).

#### 4.2.2 *LRRK2* models

*LRRK2* knockout rats significantly resist LPS- and α-syn-induced dopaminergic neurodegeneration by inhibiting the activation and recruitment of pro-inflammatory microglia in the SN (192). In *LRRK2* knockdown murine microglia, LPS treatment reduces TNF-α, inducible nitric oxide synthase, IL-1β, and IL-6 mRNA and/or protein levels, indicating LRRK2 positively regulates inflammatory induction (193).

In G2019S *LRRK2* knock-in mice, LPS-induced inflammation leads to increased iron accumulation and enhanced ferritin staining in microglia (194). Additionally, in microglia, LRRK2 exerts a negative regulatory effect on α-syn clearance by downregulating the early endocytosis pathway, as *LRRK2*-knockout microglia exhibit enhanced α-syn uptake and clearance associated with increased Rab5-positive early endosomes and improved Rab5-dynamin 1 coordination (195).

#### 4.2.3 *PINK1* models

Knocking out the *PINK1* gene results in mitochondrial dysfunction and increased vulnerability of dopaminergic neurons, and the *PINK1* model is often used to study the role of mitochondrial dysfunction in PD. In the asymptomatic phase (4 months of age), the *PINK1* knockout rat model has already exhibited mitochondrial dysfunction-related changes such as metabolic abnormalities, complex I activity-related alterations, and changes in oxygen consumption (196). In *PINK1*^-^/^-^ mouse embryonic fibroblasts, ferric ammonium citrate treatment induces the upregulation of the iron reductase STEAP2 and nucleotide surveillance-related factors. Cells lacking *PINK1* exhibit a stronger response to iron overload, and the mRNA levels of the iron storage proteins FTH1 and FTL1 are significantly increased, indicating that the iron metabolic response is altered (197).

## 5 Ferroptosis in clinical trials of PD

Growing evidence underscores the significant role of ferroptosis in the pathology of PD. This mechanistic insight has prompted the exploration of ferroptosis as a therapeutic target, given its potential contribution to dopaminergic neuronal loss and disease progression. As a result, an increasing number of clinical trials have been designed to evaluate pharmacological strategies.

### 5.1 Clinical trials of iron chelator

DFP, a blood-brain barrier (BBB)-permeable iron chelator, selectively lowers iron concentrations in PD-affected brain regions with iron overload. A preliminary clinical study showed that oral administration of DFP at a daily dose of 30 mg/kg can delay the deterioration of motor function in early-stage PD patients, with the drug showing a controllable safety profile (198). Additionally, a phase II clinical trial (25 early-stage PD patients and 12 controls; randomized, double-blind, placebo-controlled design) reported that short-term treatment with DFP was safe, with no severe systemic iron disturbances observed. The treatment was associated with reduced iron levels in the caudate and dentate nuclei, where the ferritin/T2* ratio showed a significant inverse correlation with disease duration (caudate nucleus: r = –0.536, p = 0.005; dentate nucleus: r = –0.479, p = 0.015). These findings underscore the need for future long-term clinical trials to comprehensively assess the neuroprotective potential of DFP in PD (199).

Although the phase II trial by Devos et al. confirmed that DFP effectively reduced nigrostriatal iron burden—evidenced by an increase of 1.8 ms in T2* relaxation time of the right substantia nigra compared to 0.0 ms in the placebo group (standardized difference 0.41, 95% CI 0.10–0.72)—this iron depletion was accompanied by a paradoxical worsening of clinical outcomes. After 36 weeks, patients receiving DFP showed a markedly greater increase in Movement Disorder Society Unified Parkinson’s Disease Rating Scale (MDS-UPDRS) total scores (15.6 vs. 6.3 points; difference 9.3, p < 0.001) and a higher need for dopaminergic therapy (22.0% vs. 2.7%). This deterioration may be explained by the fact that iron is not only toxic but also physiologically essential as a cofactor for TH, the rate-limiting enzyme in dopamine synthesis. Excessive chelation may deplete the iron required for dopamine production, indirectly supported by higher serum prolactin levels in the DFP group, ultimately accelerating both motor and non-motor symptom progression (200).

### 5.2 Clinical trials of interventions targeting endogenous antioxidant defenses

Enhancing endogenous antioxidant defenses, particularly the GSH system, is another strategy to combat ferroptosis. A clinical trial involving 42 PD patients (2:1 randomized to NAC or control) demonstrated that a 3-month NAC regimen (50 mg/kg intravenous weekly plus 600 mg oral twice daily, skipping oral doses on infusion days) significantly increased dopamine transporter binding compared with the control group. Dopamine transporter binding rose by 0.092 in the caudate (95% CI: 0.013–0.170, P = 0.023) and 0.135 in the putamen (95% CI: 0.056–0.214, P = 0.001), whereas the control group showed nonsignificant declines. Clinically, NAC treatment improved UPDRS scores, with a mean total decrease of 4.29 (95% CI: −6.10 to −2.47, P < 0.001), driven by both motor (−2.88, P = 0.003) and non-motor (−1.41, P = 0.01) improvements, while the control group exhibited a nonsignificant increase (+2.36, P = 0.071). Importantly, the rise in putamen dopamine transporter binding correlated strongly with UPDRS improvement (Spearman r = −0.493, P = 0.001), suggesting that NAC may restore dopaminergic integrity and function in PD (201). In a 4-week open-label study of 5 PD patients and 3 healthy controls, high-dose oral NAC (6000 mg/day) markedly enhanced peripheral antioxidant capacity but failed to elevate brain GSH levels. In PD patients, the whole-blood GSH/GSSG ratio increased by 231% and red blood cell catalase activity by 215% (vs. 18% and 109% in controls, both P < 0.05), alongside significant rises in plasma cysteine (Cmax: 15.1 μg/mL in PD vs. 25.7 μg/mL in controls). However, 3 of 5 PD patients experienced adverse effects, and 4 of 5 showed worsening UPDRS total scores (mean 32.6 → 36.6), suggesting potential tolerability or dosing concerns (202).

Intravenous or intranasal GSH supplementation is intended to enhance antioxidant capacity. A series of placebo-controlled, randomized, double-blind clinical trials investigating intravenous or intranasal administration of GSH showed improvements in motor symptoms, but these improvements lacked statistical significance compared to placebo (203–205).

Early studies suggested CoQ_10_ supplementation might slow functional decline in PD patients (206). However, subsequent larger-scale trials produced inconsistent results. A randomized phase III clinical trial involving 600 early PD patients found no evidence of clinical benefit with high-dose CoQ_10_ (1200 mg/d and 2400 mg/d) supplementation (207). Similarly, another trial confirmed the safety of CoQ_10_ at high doses but failed to show statistically significant improvements (208). In contrast, a pilot trial by Yoritaka et al. using a lower dose (300 mg/day) demonstrated significant symptomatic improvements in PD patients with wearing-off. After 48 weeks of intervention, the ubiquinol-10 group showed a mean decrease of 4.2 ± 7.5 in “on-phase” total UPDRS scores, while the placebo group had a mean increase of 2.9 ± 8.9 in scores; this between-group difference was statistically significant (t-test, p = 0.018; repeated measures ANOVA, p < 0.05) (209). These findings suggest that dose and disease stage may critically influence therapeutic outcomes.

A phase II trial by Taghizadeh et al. involving 60 PD patients (30 in intervention, 30 in placebo) demonstrated that oral co-administration of 1000 mg omega-3 fatty acids plus 400 IU vitamin E over 3 months led to a significant improvement in UPDRS total scores (−3.3 ± 10.0 vs. placebo’s +4.4 ± 14.9, P = 0.02) and a marked increase in plasma GSH concentrations (+41.4 ± 80.6 μmol/L vs. placebo’s −19.6 ± 55.9 μmol/L, P = 0.001) (210). Diacetyl-bis(4-methyl-3-thiosemicarbazonato) copperII (CuII(atsm)), an anti-ferroptotic compound that inhibits lipid radical propagation similarly to liproxstatin-1, rather than preventing iron oxidation, and is able to cross the BBB, has shown promising results in phase I PD clinical trials by improving disease severity (211).

## 6 The potential of ferroptosis in PD early diagnosis

It is estimated that by the time motor deficits are detected and individuals with PD receive a clinical diagnosis, they may have lost 30–50% of their dopaminergic neurons, accompanied by a 50–60% reduction in striatal dopamine (212), underscoring the critical need for early diagnosis in PD treatment. In this context, ferroptosis has garnered increasing attention as a promising target for early diagnostic strategies, given that iron accumulation in the SN is a characteristic feature of PD.

### 6.1 Neuroimaging biomarkers of ferroptosis in early PD

Advanced imaging modalities provide non-invasive means to assess iron deposition and evaluate the integrity of specific neuronal populations, offering invaluable insights for the early diagnosis of PD. Structural MRI is commonly used to assess iron accumulation in the SN. In assessing early-stage PD, a consistent finding on brain MRI is reduced signal intensity and atrophy in the SN and putamen (213, 214). NM-sensitive MRI can detect PD at an early stage, showing 89% sensitivity and 85% specificity (215).

QSM has emerged as a powerful MRI technique for quantifying iron content in the brain by measuring the magnetic susceptibility differences between tissues. Significant iron accumulation is evident in the SNpc of early-stage PD patients. In both the entire SNpc and its dorsolateral subregion containing nigrosome 1, QSM values were markedly higher in PD patients, with iron accumulation being more pronounced in the dorsolateral SNpc (216). This finding suggests that QSM detection targeting the dorsolateral SNpc is more specific for the pathological identification of early-stage PD. The QSM values were significantly correlated with disease duration, UPDRS II scores, and levodopa-equivalent daily dosage (217).

While not directly measuring iron content, diffusion tensor imaging parameters show alterations in the SN of PD patients that may reflect microstructural changes associated with ferroptosis-induced cell death. In early PD patients, a decrease in fractional anisotropy is commonly observed in the SN region (especially the SNpc), often accompanied by an increase in mean diffusivity or both (218, 219).

Transcranial sonography is another non-invasive imaging technique used to detect hyperechogenicity in the SN, which is associated with increased iron level**s**. Animal research has demonstrated that SN hyperechogenicity is correlated with elevated iron levels and reduced NM levels within the SN (220). The high diagnostic value of SN hyperechogenicity in differentiating PD from other movement disorders such as multiple system atrophy, essential tremor, and progressive supranuclear palsy, as well as the auxiliary role of lenticular hyperechoic area in differential diagnosis, suggest that transcranial sonography can serve as an effective discriminant index for PD diagnosis (221).

### 6.2 Hematological and molecular biomarkers related to ferroptosis

Beyond brain imaging techniques, hematological biomarkers linked to ferroptosis hold substantial potential for the early identification of PD. 8-hydroxy-2’-deoxyguanosine is a widely recognized biomarker of oxidative stress. Studies have shown that its concentrations are markedly elevated in both CSF and blood of PD patients, particularly in people living without dementia individuals, compared to healthy controls (222, 223).

The ongoing improvement in diagnostic and treatment measures for PD also includes genetic testing for risk genes for mendelian forms as well as genes related to ferroptosis. Furthermore, several microRNAs, including miR-124-3p, miR-30b-5p, miR-419-3p, and miR-214, which are linked to ferroptosis, show promise as diagnostic biomarkers for PD, offering an additional avenue for early detection (224).

## 7 Targeting ferroptosis: a therapeutic frontier for PD

**Figure 4:**
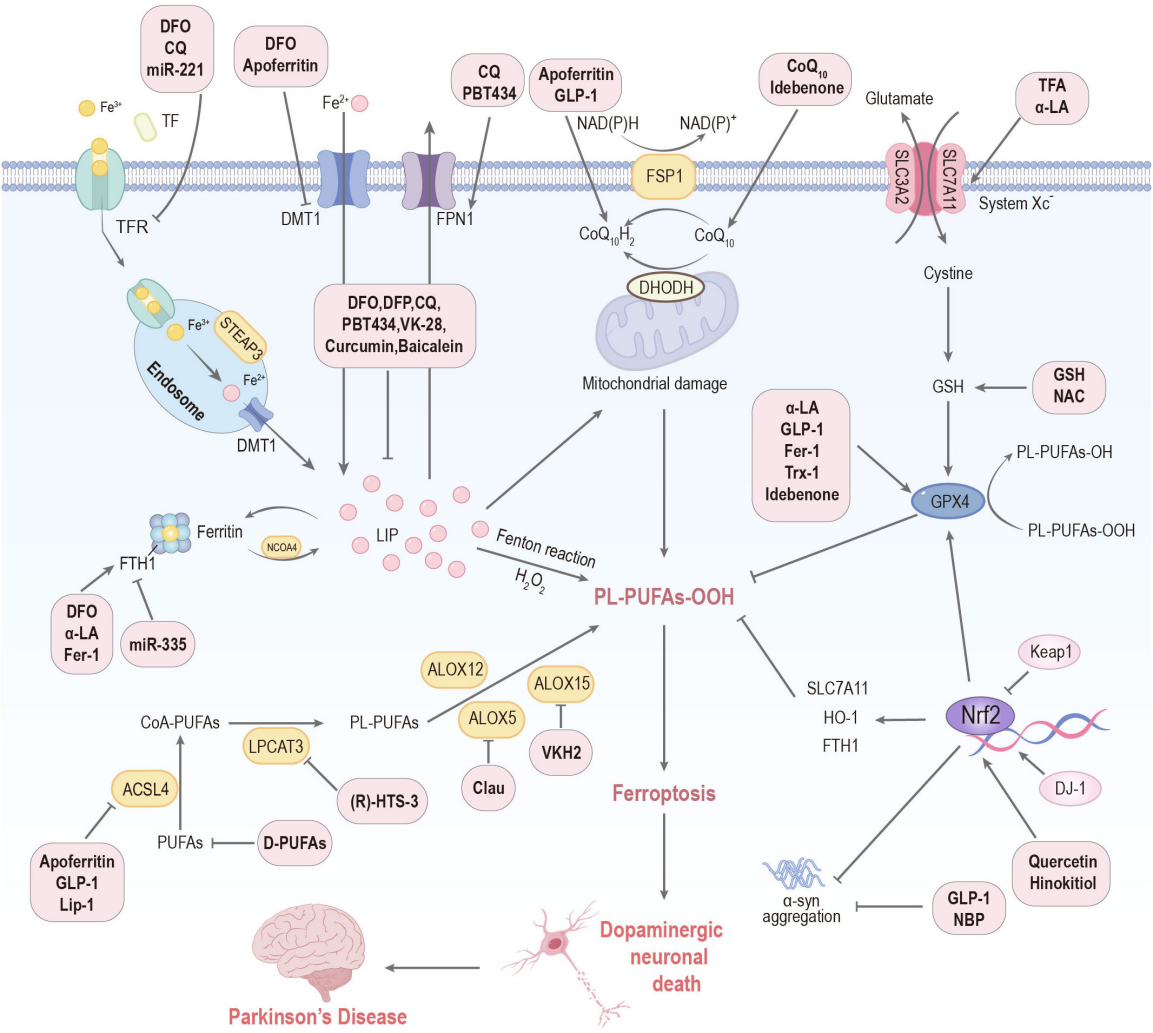
Targeting ferroptosis represents a therapeutic frontier for PD. DFO: deferoxamine; DFP: deferiprone; CQ: clioquinol; NAC: N-acetylcysteine; Clau: clausenamide; NBP: Dl-3-n-butylphthalide; Th A: thonningianin A; GLP-1: glucagon-like peptide-1; α-LA: α-lipoic acid; GSH: glutathione; VKH2: hydroquinone; Fer-1: ferrostatin-1; Lip-1: liproxstatin-1; Trx-1: thioredoxin-1; TFA: total flavonoids of Astragalus membranaceus; D-PUFAs: D-series polyunsaturated fatty acids; Keap1: Kelch-like ECH-associated protein 1; HO-1: heme oxygenase-1; FTH1: ferritin heavy chain 1; SLC7A11: solute carrier family 7 member 11; Tf: transferrin; TFR: transferrin receptor; DMT1: divalent metal transporter 1; FPN1: ferroportin 1; LIP: labile iron pool; STEAP3: ferrireductase; NCOA4: nuclear receptor coactivator 4; PL-PUFAs-OOH, phospholipid-bound polyunsaturated fatty acid hydroperoxides; PUFAs: polyunsaturated fatty acids; ACSL4: acyl CoA synthase long-chain member 4; LPCAT3: lysophosphatidylcholine acyltransferase 3; ALOX: lipoxygenase; GPX4: glutathione peroxidase 4.

Therapeutic strategies targeting ferroptosis aim to reduce intracellular iron levels and decrease oxidative stress, thereby mitigating neurodegeneration and its associated complications. These approaches encompass various mechanisms, including regulating iron homeostasis, inhibiting lipid peroxidation, modulating GPX4 and FSP1 pathways, and utilizing a range of novel compounds and natural products (**Figure 4**).

### 7.1 Targeting iron accumulation regulators

Iron chelators are a primary focus, among which DFP has been extensively investigated. Two studies showed that DFP decreased iron accumulation in the SN and slowed dyskinesia progression in early PD patients (198, 199). However, a recent large-scale phase II trial found DFP treatment was linked to worsening motor symptoms. This unexpected outcome was hypothesized to result from DFP’s inhibition of TH and, crucially, the absence of concomitant levodopa treatment in the enrolled patients, which may have potentially exacerbated dopamine deficiency. Despite this, patients treated with DFP showed improvements in brain volume (200).

Despite its lower BBB penetration compared to DFP, DFO can reduce iron accumulation in the SN, protect dopaminergic neurons, and alleviate motor dysfunction (198). Studies have shown that DFO (100 µM) can inhibit ROS accumulation and cell death induced by the ferroptosis inducer erastin (21). Intranasal administration of DFO also shows promise for improving drug delivery to the central nervous system (225).

Recently Lei et al. constructed multifunctional DFO nanomedicine (BDPR NSs) by using black phosphorus nanosheets as antioxidants and carriers to load DFO, and covalently coupling with the brain-targeting peptide RVG29. Based on receptor-mediated targeting, BDPR NSs can effectively cross the BBB, target dopaminergic neurons, specifically clear ROS and reduce iron aggregation in PD lesions, and exhibit good neuroprotective effects in PD cell, nematode, and mouse models (226).

Clioquinol, a halogenated 8-hydroxyquinoline derivative, has been found to regulate iron homeostasis in tissues by effectively chelating Fe^2+^, thereby inhibiting ferroptosis (227). In MPTP-induced monkey model of PD, clioquinol significantly alleviated both dyskinesia and non-motor impairments, concomitant with a reduction in iron accumulation and ROS levels in the SN (228).

Finkelstein et al. found that PBT434, a novel compound with moderate iron affinity, can protect the survival of SNpc neurons, improve nigrostriatal connectivity and motor function in mice, without disrupting normal iron homeostasis (229). With iron-chelating properties and monoamine oxidase inhibitory activity, VK-28 derivatives (including M30, M32, and HLA20) target multiple neurodegenerative pathways—they suppress oxidative stress-related pathways (e.g., Keap1-Nrf2), modulate the AKT/mTOR pathway, and alleviate α-syn aggregation (230). As a novel bifunctional iron chelator, SK4 is structurally based on DFP. It innovatively integrates a high-affinity iron-chelating group and an L-type amino acid transporter 1 transporter-targeting structure, and has demonstrated significant neuroprotective effects in a variety of PD-related neurotoxicity models (231).

Beyond traditional chelators, apoferritin regulates ferroptosis through its effect on proteins involved in iron metabolism, including ACSL4 and FSP1 (230). Iron carbonyl compounds, such as iron pentacarbonyl, have been shown to fine-tune iron utilization to prevent neuronal damage *in vivo*. Paradoxically, while iron toxicity in PD is linked to dysfunctional iron metabolism, intragastric delivery of iron carbonyl boosts the biosynthesis of iron-sulfur clusters and elevates aconitase 1 activity, which is crucial for mitochondrial function and iron homeostasis, thereby preventing ferroptosis *in vivo* (188). Therefore, the key issue in PD may lie in the dysregulation of iron utilization rather than excessively high iron levels per se. Accordingly, therapeutic strategies for PD might not only aim at lowering iron levels, but also consider regulating iron utilization, for instance by strictly controlling iron absorption.

### 7.2 Targeting system Xc^-^/GPX4 pathway

As a GSH precursor, NAC inhibits ferroptosis by increasing GSH levels and cooperating with GSH-dependent enzymes to target and neutralize toxic lipids derived from ALOX5 (232). Monti et al. found that NAC administration via a combination of intravenous injection and oral route could increase the levels of dopamine transporters in the striatum of PD patients as well as improve their motor function (201, 233).

The coadministration of NAC and DFP attenuated iron accumulation and mitochondrial dysfunction, and improved synaptic plasticity and cognition in iron-fed rats (234). In 2020, Mursaleen et al. successfully formulated NAC alone and in combination with DFO into Pluronic F68 and dequalinium nanocarriers for neuronal delivery for the first time. The results demonstrated that these nanocarriers possess characteristics enabling them to access the brain, and they are at least as effective as the corresponding free drugs in protecting against rotenone-induced reduction in cell viability, increased iron content, and oxidative stress. Among them, 1000μM Pluronic F68 + dequalinium NAC showed the strongest effect, suggesting that NAC alone might be more suitable (235). However, further evaluation of their brain penetration ability using a cellular BBB model is required to advance preclinical testing.

GSH supplementation, particularly intravenously, enhances antioxidant potential and could exert a delaying impact on neurodegenerative processes in PD (236). However, clinical trials investigating intravenous or intranasal GSH showed improvements in PD symptoms but lacked statistical significance over placebo, suggesting a higher dose might be required (203–205). Selenium enhances the body’s antioxidant capacity by participating in the synthesis of various selenoproteins, such as the glutathione peroxidase and thioredoxin reductase families. Selenium supplementation can further boost the activity of GPX4 and other antioxidant systems, thereby exerting protective effects (237, 238). Selenium supplementation partially reverses MPTP-induced impairment of dopaminergic neurotransmission (239). In a PQ-induced PD model, selenium helped maintain locomotor activity and leukocyte DNA integrity (240). Additionally, thioredoxin-1, a core component of the thioredoxin system, also contributes to these protective effects by maintaining cellular redox balance (237, 238).

### 7.3 Targeting FSP1/CoQ_10_ pathway

Intrastriatal administration of CoQ_10_ showed higher efficacy in exerting neuroprotective effects than oral CoQ_10_ delivery in a rat PD model, requiring 17,000 times lower dose (241). Although Shults et al. found a positive result (206), subsequent clinical studies on long-term supplementation of CoQ_10_ have shown that high dosages (1200–2400 mg/d) are safe but generally lack significant clinical benefits (207, 208). In contrast, 300 mg/d of reduced CoQ_10_ noticeably improved symptoms in specific PD populations, such as patients with the “wearing-off” phenomenon (209). This suggests that dosage, formulation, and patient subtype may affect the therapeutic efficacy of CoQ_10_, and further investigations are warranted.

DPT3f, a derivative of the recently identified ferroptosis inhibitor DPT, demonstrates the strongest anti-ferroptotic activity among its analogs, attributable to its unique molecular structure. DPT3f enhances FSP1/CoQ_10_ pathway antioxidant activity by upregulating FSP1 protein expression, promotes CoQ_10_ conversion to reduced CoQ_10_H_2_ to scavenge lipid peroxides, thus inhibiting dopaminergic neuron ferroptosis, protecting SN neurons and alleviating PD-related damage (230).

### 7.4 Radical trapping agents

CuII(atsm) exhibits pronounced neuroprotective properties. By scavenging peroxynitrite, it attenuates peroxynitrite-mediated nitration and aggregation of α-syn, thereby mitigating SN dopaminergic neuronal loss and preserving dopamine metabolism. As a result, CuII(atsm) improves motor performance and cognitive function in PD models, including MPTP- and 6-OHDA-induced mice as well as transgenic mice overexpressing the human A53T α-syn mutation (242). In a phase I clinical trial, CuII(atsm) administration was linked to a marked decrease in disease severity in PD patients (211).

Carnosine conjugates lipid peroxides to provide oxidative protection (243), and has also shown potential, as carnosine supplementation resulted in potential symptomatic benefits in a clinical trial enrolling patients with PD (244). A study has shown that combined supplementation with ω-3 fatty acids and vitamin E in PD patients resulted in a marked decrease in the UPDRS total score, a notable elevation in plasma GSH concentration, and a substantial reduction in plasma MDA levels after adjusting for baseline factors (210).

As one of the earliest discovered ferroptosis inhibitors, Fer-1 functions through targeting and scavenging initiating alkoxyl radicals, forming complexes with iron, and undergoing cyclic regeneration (245). In 6-OHDA models, Fer-1 blocks the downregulation of GPX4 and concurrent upregulation of sorting nexin 5, thereby maintaining ferroptosis resistance (246). However, Fer-1’s poor metabolic stability limits its suitability *in vivo*. Fer-1 derivatives such as SRS11–92 exhibit greater metabolic stability and effectiveness compared to Fer-1 (247, 248). Liproxstatin-1, a spiroquinoxalinamine-based compound with favorable pharmacological properties, attenuates lipid peroxidation by suppressing lipid ROS generation, lowering MDA and 4-HNE levels, and downregulating ACSL4 expression (249).

### 7.5 Targeting enzymes in lipid peroxidation

Targeting enzymes crucial for lipid peroxidation synthesis such as ACSL4 and LPCAT3 is a potential therapeutic approach (250). PRGL493, as an ACSL4 inhibitor, inhibits the growth of breast and prostate tumors and reduces therapeutic resistance by targeting ACSL4 (251). The potent LPCAT3 inhibitor, (R)-HTS-3, demonstrated partial protection against ferroptosis (252). Clausenamide (Clau) has been shown to protect dopaminergic neurons by directly inhibiting ALOX5 activation and its nuclear translocation, thereby reducing lipid peroxidation and suppressing ferroptosis (253).

The calcium-independent phospholipase A2β (iPLA2β) exerts a neuroprotective effect by selectively hydrolyzing the ferroptotic signal molecule 15-HpETE-PE, while its functional defects such as mutations or reduced expression represent key contributors to PD pathogenesis (254). Deficiency of iPLA2β results in reduced docosahexaenoic acid (DHA) levels, elevated pro-inflammatory factors, and decreased levels of neuroprotective BDNF, thereby triggering microglial activation and inflammatory responses. DHA supplementation can partially reverse these effects. Also, DHA can alleviate motor dysfunction in PLA2G6 mutant models, and reduce neuroinflammation and lipid peroxidation (255).

### 7.6 Natural plant product

Flavonoids such as curcumin, baicalein, and quercetin exhibit iron-chelating, antioxidant, and anti-inflammatory effects. Curcumin exerts a neuroprotective effect on nigral dopaminergic neurons in a 6-OHDA-injected PD rat model by chelating iron to suppress iron-induced degeneration. This is evidenced by its ability to reduce the number of iron-stained cells in the SN, reverse the loss of dopamine and TH-positive neurons, and its mechanism is linked to iron chelation-mediated reduction of oxidative stress (256). Baicalein protects SK-N-MC neuroblastoma cells from menadione-induced oxidative damage and apoptosis by chelating free iron, scavenging ROS, and modulating apoptotic pathways (257). Quercetin, a widely distributed flavonoid, significantly suppresses ferroptosis through the activation of the Nrf2 protein (258).

Paeoniflorin, a monoterpene glycoside, protects dopaminergic neurons against MPP^+^-induced toxicity by inhibiting ferroptosis, as evidenced by reduced lipid ROS accumulation and restored GPX4 expression. Studies have demonstrated that paeoniflorin activates the Akt/Nrf2/GPX4 signaling cascade, thereby facilitating Nrf2 nuclear translocation and subsequent GPX4 upregulation to inhibit iron-dependent lipid peroxidation (259).

Thonningianin A, an ellagitannin from *Thonningia sanguinea* Vahl, protects dopaminergic cells from 6-OHDA-induced ferroptosis. Mechanistically, it activates the Nrf2 cytoprotective system by promoting Keap1 degradation via Atg7-dependent autophagy, disrupting the Keap1-Nrf2 interaction and enhancing Nrf2 nuclear translocation (260). Hinokitiol, a natural compound extracted from *Chamaecyparis taiwanensis*, potently inhibits ferroptosis to alleviate neuronal damage *in vitro* and *in vivo*. It chelates iron and activates Nrf2, upregulating genes encoding SLC7A11, GPX4, and HO-1 to suppress lipid peroxidation and iron accumulation (261).

### 7.7 Other regulators

Glucagon-like peptide-1 (GLP-1) receptor agonists, a class of medications commonly used to treat diabetes and obesity, have exhibited promising benefits for PD patients (262). The probiotic strain *Lactococcus lactis* MG1363-pMG36e-GLP-1 exerts significant neurotrophic effects on MPTP-induced PD mouse models, and its mechanism is mainly achieved through inhibiting ferroptosis, alleviating oxidative stress, improving intestinal microbiota imbalance, and enhancing intestinal barrier integrity (263). In experimental PD models, GLP-1 receptor agonists (GLP-1RAs) can ameliorate the progression of α-syn pathology and dopaminergic neuronal loss, probably through anti-neuroinflammatory and neuroprotective effects (264). In patients with early PD, the GLP-1RA lixisenatide can modestly slow the progression of motor dysfunction over 12 months of treatment, but it is associated with gastrointestinal side effects, and its long-term efficacy and safety require further verification (265).

Lapatinib ditosylate, an approved anti-cancer drug, provides neuroprotective benefits in rotenone-induced PD rat models and holds promise for repurposing as a disease-modifying agent for PD. Lapatinib ditosylate can activate the Nrf2/GPX4/GSH axis and inhibit the accumulation of 4-HNE, thereby alleviating motor dysfunction and neuropathological damage (266).

## 8 Conclusion and future perspectives

PD, as a complex neurodegenerative disorder, remains incompletely understood in terms of its precise pathogenic mechanisms. Emerging evidence suggests that ferroptosis is crucially involved in the onset and progression of PD. This review systematically summarizes the definition of ferroptosis and its intricate regulatory network, including iron metabolism imbalance, lipid peroxidation, and dysfunction of antioxidant systems. We further discuss how PD-specific pathological features, such as aberrant aggregation of α-syn and degenerative loss of dopaminergic neurons, sensitize neural cells to ferroptosis. Evidence from animal models and *in vitro* experiments provides substantial support for the contribution of ferroptosis to PD pathogenesis.

Although numerous preclinical studies have demonstrated significant therapeutic potential of targeting ferroptosis pathways—such as using iron chelators, modulating GPX4 activity, or inhibiting lipid peroxidation—recent clinical trial results indicate that strategies aiming solely to reduce iron accumulation may have limited efficacy in clinical practice. These findings suggest that more precise intervention timing, personalized treatment approaches, and combinatorial strategies may be required to achieve better outcomes.

Looking ahead, research on ferroptosis in the context of PD faces both opportunities and challenges. First, it is essential to unravel in depth the causal association and temporal sequence between ferroptosis and neurodegeneration, as well as to explore cell type-specific roles of iron accumulation and ferroptosis across various neural populations. Second, the development of sensitive and specific biomarkers of ferroptosis is crucial for early diagnosis, disease progression monitoring, and therapeutic evaluation in PD. From a therapeutic perspective, future efforts should focus on identifying and validating novel ferroptosis regulators, particularly those targeting non-canonical pathways. Integrating patient-specific genetic and metabolic profiles could also facilitate the design of individualized, multi-target intervention strategies. In addition, cutting-edge technological approaches, including gene editing and non-coding RNA modulation, hold promise for precise regulation of ferroptosis. At the same time, ferroptosis research should be integrated with studies on other central pathological processes in PD, including mitochondrial dysfunction and neuroinflammation, to investigate the interplay among multiple cell death pathways and develop combinatorial neuroprotective and disease-modifying therapies. Notably, natural compounds as potential ferroptosis inhibitors warrant further systematic investigation and high-quality clinical validation to confirm their safety and efficacy.

In conclusion, ferroptosis represents a key mechanism in PD pathogenesis, offering new perspectives for understanding this complex disease and highlighting promising avenues for developing safer and more precise diagnostic and therapeutic strategies. Through continued exploration of its molecular underpinnings, the discovery of reliable biomarkers, and the advancement of innovative treatment modalities, we may ultimately provide more effective interventions for patients with PD and generate valuable insights applicable to other neurodegenerative disorders.
